# Whole-genome assembly and annotation of the bumblebee wax moth, *Aphomia sociella*

**DOI:** 10.1093/g3journal/jkaf281

**Published:** 2025-11-27

**Authors:** Ronja Marlonsdotter Sandholm, Gustav Vaaje-Kolstad, Sabina Leanti La Rosa

**Affiliations:** Faculty of Chemistry, Biotechnology and Food Science, Norwegian University of Life Sciences,1432, Ås, Norway; Faculty of Chemistry, Biotechnology and Food Science, Norwegian University of Life Sciences,1432, Ås, Norway; Faculty of Chemistry, Biotechnology and Food Science, Norwegian University of Life Sciences,1432, Ås, Norway

**Keywords:** bumblebee wax moth, *Aphomia sociella*, genome assembly, annotation, phylogeny

## Abstract

The bumble bee wax moth, *Aphomia sociella*, is an important lepidopteran pest impacting bee colonies essential for pollination and apiculture. Like other moths, this species has been reported to ingest plastics. Although specific enzymes have been proposed to facilitate plastic catabolism in some moths, with controversial results, this remains entirely unexplored in *A. sociella*. Despite the biological and ecological relevance of *A. sociella*, sequence efforts aimed at understanding the genetic makeup of this species have not yet been undertaken. In this work, we successfully achieved a high-quality de novo genome assembly of *A. sociella* and comprehensive gene annotations generated from long-read DNA and RNA sequencing with Oxford Nanopore Technology. The haploid assembly includes 347 contigs, with an N50 of 4.96 Mb, and contains 12,618 protein-coding genes. Benchmarking Universal Single-Copy Orthologs (BUSCO) analyses indicates that the assembly has a high level of completeness (98.8%) and low level of fragmentation (4.1%) and duplication (0.2%). Phylogenomic analyses with other members of the Lepidoptera order placed *A. sociella* in the same clade as *Aphomia cephalonica* and indicates close evolutionary relationships with the other two species in the subfamily *Galleriinae*, namely *Achroia grisella* and *Galleria mellonella*. This new high-quality genome assembly, and associated annotations, represents a valuable resource for investigating the genomic basis of ecological specialization of *A. sociella*, including wax and possibly plastic utilization, while offering critical support for research aimed at developing sustainable and effective pest management strategies.

## Introduction

In recent years, sequencing the genomes of all living species has emerged as a cornerstone goal in biodiversity science. Initiatives like the Earth BioGenome Project aim to generate high-quality reference genomes for all eukaryotic life on Earth, creating a comprehensive molecular archive of Earth's biological diversity (Lewin et al. 2022). These reference genomes serve as foundational resources that empower a wide range of scientific and practical applications, including studies of evolution and gene function, conservation genetics and species breeding programs. Moreover, for species that pose serious economic threats to agriculture and farming, elucidating the underlying genetic determinants can facilitate the development of novel, targeted control strategies.

The order Lepidoptera consists of butterflies and moths, with around 160,000 described species (van Nieukerken et al. 2011). This makes the order one of the largest in the animal kingdom, exceeded only by Hymenoptera and Coleoptera (Forbes et al. 2018). Lepidoptera includes the family *Pyralidae*, of which the wax moths belong. To date, 32 out of 6,440 species in this family have publicly available reference genomes (Schoch et al. 2020; Khramov 2025). This family of moths is a diverse group, with many members being economically important pests, infesting key agricultural products, such as dried fruits and vegetables, grains, and seeds. Within this family, the subfamily *Galleriinae* includes notable species such as the bumblebee wax moth *Aphomia sociella*, the greater wax moth *Galleria mellonella* and the lesser wax moth *Achroia grisella*. While wax moth larvae are pests of beehives, *A. sociella* primarily attacks bumblebee nests but can also invade weakened commercial honeybee colonies (Krams et al. 2025). Females deposit their eggs within the nests, where the larvae feed on wax comb and honey, causing severe damage that can lead to colony collapse. Given that bumblebees play a crucial commercial role in agriculture by providing essential pollination services for many crops across temperate regions of the Northern Hemisphere, moth infestations pose significant environmental threats and economic challenges that profoundly impact beekeeping sustainability.

In addition to their role as pests, several moths, including *A. sociella*, have been reported to consume plastics. In recent years, *G. mellonella* and *A. grisella* have gained particular interest for its “plastivore” lifestyle and has become increasingly studied as a potential source of plastic degrading enzymes, allegedly originating both from the insect itself and from its endogenous gut microbiome (Ren et al. 2019; Zhang et al. 2020). Despite interest in the plastic degrading abilities of insect larvae, the evidence remains controversial, as studies have reported inconsistent results regarding plastic oxidation or bioassimilation (Réjasse et al. 2022; Stepnov et al. 2024).

Control strategies for managing moth populations have included the use of pheromone-baited traps and bioinsecticides composed of lethal viral or bacterial pathogens targeting the larval stages (Zhang et al. 2021; Niu et al. 2024). Pheromones are release by both males and females to attract each other during mating (Kindl et al. 2011 , 2012). Targeting pheromones offers an effective approach for selective pest control is due to their species specificity, high potency in very small amounts, and low toxicity to non-target animals (Witzgall et al. 2010). While there is a limited understanding of the identity, genetic basis, and regulation of pheromone production in *A. sociella*, such knowledge could be applied to enhance the efficacy of pheromone-baited traps and bioinsecticides to control the bumblebee wax moth populations.

Here, capitalizing on advancements in long-read sequencing technology, we present a high-quality genome assembly of *A. sociella*. We obtained gene annotations through short- and long-read RNA sequencing data and demonstrated the utility of our new genome assembly through comparative genomics. We envisage that this genome resource will facilitate functional studies into the moth, giving a deeper understanding of basic biological mechanisms, such as life cycle and survival, pheromone signaling and immunity that may be of value for developing pest control measures.

## Methods

### Tissue collection

*A. sociella* larvae were collected from a private breeder of tree bumblebees (*Bombus hypnorum*) in Enger, Innlandet county, Norway. The collection occurred on the 16^th^ of September 2022. The larvae were fed remnants of the infested bumblebee nest until they arrived in the laboratory at the Norwegian University of Life Sciences. Note that the sex of the specimen could not be determined at the larval stage. Larvae were rinsed in sterile MilliQ water and either stored at −20 °C before DNA extraction or preserved in RNA*later* Stabilization solution (Invitrogen, Cat. no.: AM7020) at −80 °C before RNA extraction.

### DNA extraction and whole-genome sequencing

High molecular-weight (HMW) DNA from an individual larva was extracted using a previously established protocol, with minor modifications (Mugford et al. 2020). The frozen larva was briefly minced on ice before homogenization in 750 µL cold phosphate-buffered saline (PBS, Sigma-Aldrich, Cat. no.: P4417) using a TissueRuptor II (QIAGEN, ID: 9002755), with a disposable probe (QIAGEN, ID: 990890) at 35,000 rpm for 10 s. The homogenized tissue was transferred to a 2 mL Eppendorf tube and centrifuged at 3,000*×g* for 5 min at 4 °C. The resulting pellet was resuspended in 1 mL of cold PBS and subjected to the cetrimonium bromide:chloroform method to obtain HMW DNA. Briefly, the 1 mL sample was mixed with 100 µL cetrimonium bromide buffer (2% w/v CTAB, 1.4 M NaCl, 20 mM EDTA, and 100 mM Tris-HCl pH 8.0) and incubated overnight at 50 °C. Five microliters of RNAse A (0.2 mg/mL) was added and the solution was incubated at 37 °C for 15 min to remove RNA. The aqueous phase was recovered after inversion mixing 100 µL chloroform for 10 min followed by centrifugation at 5,000*×g* for 15 min to separate the phases. One hundred microliters of ice-cold isopropanol was added to the recovered aqueous phase in a drop-wise fashion and mixed by inversion before incubation at −20 °C for 2 h. HMW DNA was pelleted by centrifugation at 15,000*×g* for 20 min at 4 °C, and the supernatant was discarded. The DNA was washed with 0.5 mL room-temperature 75% ethanol, centrifuged for 5,000*×g* for 20 min, decanted, and air-dried before resuspension in 20 µL nuclease-free water. The resulting DNA was quantified using the Qubit Broad Range DNA assay (Invitrogen), according to the manufacturer's protocol, on a Qubit 1.0 fluorometer. DNA quality was assessed using a NanoDrop One UV-Vis spectrophotometer (Thermo Scientific, Wilmington, USA). The sequencing library was prepared using the Ligation Sequencing kit SKQ-LSK110 (Oxford Nanopore Technologies) following the manufacturer's instructions. The library was loaded for sequencing on a single R9.4.1 flow cell on a PromethION platform (Oxford Nanopore Technologies (ONT)) at the Norwegian University of Life Sciences. Sequencing was conducted for 72 h using default voltage and temperature settings. The system was operated with the MinKNOW v22.10.5 software.

### RNA extraction and sequencing

A whole single larva of *A. sociella* was used for RNA extraction. RNA was obtained by homogenizing the tissue in 750 µL PBS with a TissueRuptor II (QIAGEN, ID: 9002755) with a disposable probe (QIAGEN, ID: 990890) at 35,000 rpm for 1 min, followed by extraction with the RNeasy Mini Kit (QIAGEN, ID: 74104) according to the manufacturer's instructions. RNA concentration and purity were determined using a Qubit 3.0 fluorometer and a NanoDrop One instrument (Thermo Scientific). RNA integrity was verified using an Agilent 4200 Tape Station System (Agilent Technologies, Santa Clara, CA, USA). PolyA-enriched RNA was sequenced on the Illumina NovaSeq 6000 platform, utilizing a pair-end 300 bp sequencing strategy. As per the manufacturer's protocol, Novogene (Beijing, China) carried out library preparations using a TruSeq Stranded mRNA kit (Illumina, San Diego, CA, USA). A long-read sequencing library of the extracted RNA was prepared using the PCR-cDNA Barcoding kit SQK-PCB111.24 (Oxford Nanopore Technologies) and sequenced on a R10.4 flow cell on a PromethION platform (Oxford Nanopore Technologies) at the Norwegian University of Life Sciences. Sequencing was conducted for 72 h using default voltage and temperature settings. The system was operated with the MinKNOW v23.07.12 and basecalling was performed using GPU-enabled Guppy v7.1.4 using the high-accuracy model.

### Genome assembly

Basecalling on the ONT raw reads was performed using GPU-enabled Guppy v6.3.8 with the high-accuracy model (Oxford Nanopore Technologies 2023). Reads were filtered by quality using fastp v0.23.4 with the --length_required 50, --qualified_quality_phred 7, and --trim_front1 400 parameters (Chen et al. 2018). Four-genome assemblies were generated using Canu v2.2 using the -nanopore-raw parameter (Koren et al. 2017), where one was obtained using standard parameters and three by adjusting the error rate for each assembly (correctedErrorRate = 0.01, correctedErrorRate = 0.02, and correctedErrorRate = 0.03). Error correction was performed using Inspector v1.0.2 (Chen et al. 2021). Quality assessment of the assemblies was conducted using Merqury v1.3 (Rhie et al. 2020) and Benchmarking Universal Single-Copy Orthologs (BUSCO) v5.8.3 with the -m geno and -l lepidoptera_odb10 parameters (Manni et al. 2021). Given that the Canu assembly with an adjusted error rate of 2% had higher contiguity (Supplementary Table 1), it was chosen for downstream processing. Purge Haplotigs v1.1.2 (Roach et al. 2018) was used to generate a primary assembly, identify haplotigs and artefactual contigs, and eventually obtain a deduplicated, non-redundant genome assembly for the insect, with the parameters for purge_haplotigs cov being -l 5, -m 100, and -h 200. The genome assembly was also screened for contaminations using FCS-GX v0.5.0 (Astashyn et al. 2024), and assessed using assembly_stats v0.1.4 (Trizna 2020), both with standard parameters.

### Genome annotation

Before annotation, repeat elements of the genomes were identified and masked using RepeatModeler v4.0 (Smit and Hubley 2015) with -database function followed by RepeatMasker v1.0 (Smit et al. 2015) using default setting for de novo prediction. Protein-coding genes were predicted using transcriptomics-based methods with short- and long-RNA reads. Short-read RNA sequences were filtered using fastp v1.0.1 (Chen 2025), and long-read RNA sequences were filtered using fastplong v0.4.1 (Chen 2023), both using standard parameters. The genome was annotated using the external NCBI Eukaryotic Genome Annotation Pipeline (EGAPx) v0.4.1-alpha (NCBI 2025), which was run using quality filtered long- and short-read RNA with standard parameters and in singularity mode (“-e singularity”). The genome was scanned for tRNAs using tRNAscan-SE v2.0.12 (Lowe and Eddy 1997) using standard parameters.

### Phylogenomic analysis

To investigate the phylogenomic relationship between *A. sociella* (this study) and other members of the Lepidoptera order, publicly available genomes were obtained and BUSCO orthologous genes (lepidoptera_odb12) aligned using OrthoFinder v3.0.1b1 (Emms and Kelly 2019). The final species tree represents the best-supported topology based on species tree inference from multi-copy gene trees, determined using STAG, and rooted using STRIDE (Emms and Kelly 2017); both programs were run with default parameters within the OrthoFinder pipeline. The genomes used for analysis included *Bombyx mori* (GCA_014905235.2), *Agriphila tristella* (GCA_928269145.1), *Micropterix aruncella* (GCA_944548615.1), *Papilio xuthus* (GCA_000836235.2), *Plutella xylostella* (GCA_932276165.1), *A. grisella* (GCA_030625045.1), *Acrobasis consociella* (GCA_963555685.1), *Acrobasis repandana* (GCA_963576875.1), *Acrobasis suavella* (GCA_943193695.1), *Amyelois transitella* (GCA_032362555.1), *Anerastia lotella* (GCA_964291865.1), *Apomyelois bistriatella* (GCA_947044815.1), *Cactoblastis cactorum* (GCA_020352625.1), *Corcyra cephalonica* (GCA_040436485.1), *Dioryctria mendacella* (GCA_964374295.1), *Elegia similella* (GCA_947532085.1), *Endotricha flammealis* (GCA_905163395.2), *Ephestia elutella* (GCA_018467065.1), *Ephestia kuehniella* (GCA_921024065.1), *Euzophera pinguis* (GCA_947363495.1), *G. mellonella* (GCA_026898425.1), *Homoeosoma sinuella* (GCA_964340625.1), *Hypsopygia costalis* (GCA_937001555.2), *Hypsopygia rubidalis* (GCA_965279855.1), *Pempelia palumbella* (GCA_964656235.1), *Phycita roborella* (GCA_964059365.1), *Plodia interpunctella* (GCA_027563975.2), *Pyralis farinalis* (GCA_947507595.1), *Pyralis regalis* (GCA_965194485.1), *Rhodophaea formosa* (GCA_963082605.1), *Synaphe punctalis* (GCA_965276045.1), and *Zophodia grossulariella* (GCA_965234215.1). Notably, the genus *Corcyra* has been synonymized with *Aphomia* (Leraut 2014). Accordingly, *Corcyra cephalonica* is hereafter referred to as *Aphomia cephalonica* throughout this work. The phylogenomic tree was generated using the packages ggtree v3.10.0 (Xu et al. 2022), aplot v0.2.2 (Yu 2023), ape v5.7.1 (Paradis and Schliep 2019), and tidyverse v2.0.0 (Wickham et al. 2019) in R v4.3.3 (R Core Team 2024).

Synteny regions were determined using minimap2 v2.26 (Li 2018), by aligning the assembled *A. sociella* genome to the publicly available genomes of *G. mellonella, A. grisella*, and *A. cephalonica* using the parameter -x asm10. To generate synteny plots, the packages tidyverse v2.0.0, ggplot2 v3.5.2 (Wickham 2016), and cowplot v1.1.3 (Wilke 2024) were used in R v4.3.3.

## Results and discussion

### Genome assembly and annotation

**Fig. 1.**
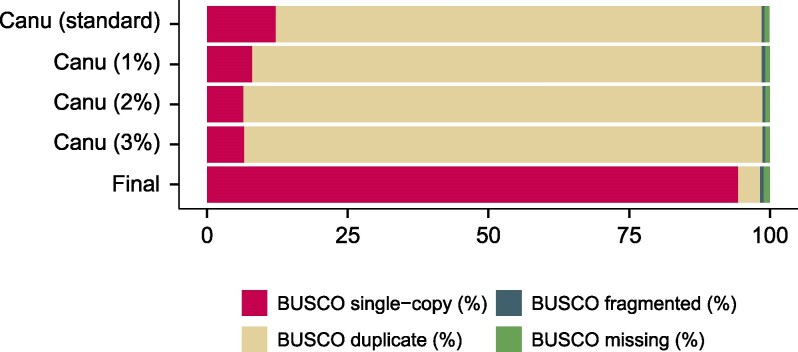
BUSCO scores of the Canu assemblies using standard parameters, and adjusted error rates of 1%, 2%, and 3%, as well as the final assembly after haplotype reassignment with Purge Haplotigs v1.1.2.

Prior to this study, no publicly available genome assembly existed for *A. sociella*. We performed whole-genome sequencing, assembly, and gene annotation by isolating genomic DNA from a single *A. sociella* larva. The genome sequence was assembled from 85.07 Gb data and 12.86 M ONT reads. Four assemblies were generated using Canu with default settings and an adjusted error rate from 1% to 3%. Assembly quality and completeness were quantitatively assessed based on evolutionarily informed expectations of gene content (Fig. 1). The Canu assembly generated with an adjusted error rate of 2% exhibited the most contiguous genome, as indicated by its highest contig N50 (2.58 Mb) when compared with the other assemblies and was therefore selected for further refinement (Supplementary Table 1).

The selected assembly consisted of 1,294 Mb, 1,451 contigs, and N50 of 2.58 Mb while displaying a 92.2% duplication rate as determined by BUSCO (Supplementary Table 1). Allelic contigs were assessed using Purge Haplotigs and 47.6% (616 Mb) of the *A. sociella* genome was reassigned as haplotigs, while 0.22% (2.9 Mb) was reassigned as artifacts. The final assembled genome had a total length of 675 Mb distributed across 347 contigs (N50 = 4.96 Mb). Based on the genome mode for BUSCO analysis, the assembly was 98.3% complete, 0.6% fragmented, and had a duplication rate of 3.9% (Fig. 1). These metrics indicate that the genome assembly of *A. sociella* is of high quality.

RepeatModeler and RepeatMasker analyses classified and then masked 55.96% of the *A. sociella* genome for repetitive elements. Retroelements represented 33.40% of the genome, including short interspersed nuclear elements (14.90%), long interspersed nuclear elements (15.34%), and long terminal repeats (3.16%). No satellite DNA was detected, and only 1.32% of the genome was represented by simple repeats. Retroelements were the most common categories of classified repeats, and some unclassified repeats (13.53%) were also detected.

Next, functional gene annotation for the genome assembly was obtained using a total of 28.1 Gb of data (28.3 M reads) from long-read RNA sequencing and 16.8 Gb of data (112.3 M reads) from short-read RNA sequencing. Annotation using EGAPx predicted a total of 13,020 genes, 14,081 mRNAs, and 473 tRNAs. A completeness of 98.8% was achieved using BUSCO completeness evaluation of genes, with 94.7% single-copy genes and a duplication rate of 4.1% (Table 1).

**Table 1.** Number of coding sequences, mRNAs, tRNAs and genes, as well as BUSCO completeness, duplications, and fragments for the annotated genome.

Feature	Annotation
Coding sequences	14,081
Number of genes	13,020
Number of protein-coding genes	12,618
Number of mRNAs	14,081
Number of tRNAs	473
BUSCO on proteins	
BUSCO completeness (%)^a^	98.8
BUSCO single copy (%)^a^	94.7
BUSCO duplicate (%)^a^	4.1
BUSCO fragmented (%)^a^	0.2
BUSCO missing (%)^a^	1.0

^a^Determined using the BUSCO lepidoptera_odb10 dataset.

### Phylogeny of *A. sociella*

**Fig. 2.**
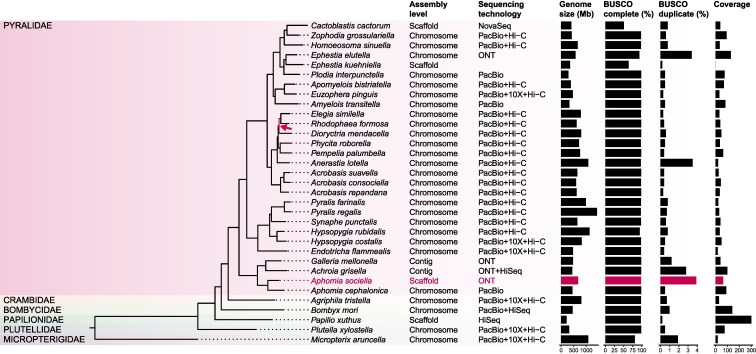
Phylogenetic placement of *A. sociella*. The tree was constructed with OrthoFinder v3.0.1b1 using BUSCO single-copy orthologs from the genome assembly generated in this study along with publicly available genomes from other species of the Lepidoptera order, of which *M. aruncella* was automatically selected as the tree root. All nodes are supported by 100% bootstrap values, except one (indicated with an arrow and pink colored node, 61.1% bootstrap). Assembly level denotes the resolution of the genome, whether it is chromosome, scaffold of contig level. The sequencing platforms used for the assemblies are Illumina NovaSeq (NovaSeq), Illumina HiSeq (HiSeq), Pacific Biosciences (PacBio), Arima2 Hi-C (Hi-C), 10X Genomics Chromium (10X) and Oxford Nanopore Technologies (ONT), or a combination. The BUSCO complete (%) and BUSCO duplicate (%) were obtained using the lepidoptera_odb12 dataset.

A set of orthologs was chosen to perform phylogenomic analysis of the assembled genome of *A. sociella* and 31 species of the order Lepidoptera (Supplementary Table 2). We recovered a robust phylogeny of the order Lepidoptera, with 28 genomes (including *A. sociella*) belonging to the family *Pyralidae*, with *B. mori* (family *Bombycidae*), *A. tristella* (family *Crambidae*), *M. aruncella* (family *Micropterigidae*), *P. xuthus* (family *Papilionidae*), and *P. xylostella* (family *Plutellidae*) included as outgroups. *A. sociella* is part of the subfamily *Galleriinae* that also includes *G. mellonella, A. grisella*, and *A. cephalonica* (Fig. 2). All nodes in the tree except one were supported with 100% bootstrap. *A. sociella* was placed in the same clade as *A. cephalonica* (rice moth). The phylogenomic relationship between these species reinforces the taxonomic classification. As expected, the closest relatives of *A. sociella* correspond to species taxonomically classified as members of the *Galleriinae* subfamily (Scholtens and Solis 2015).

**Fig. 3.**
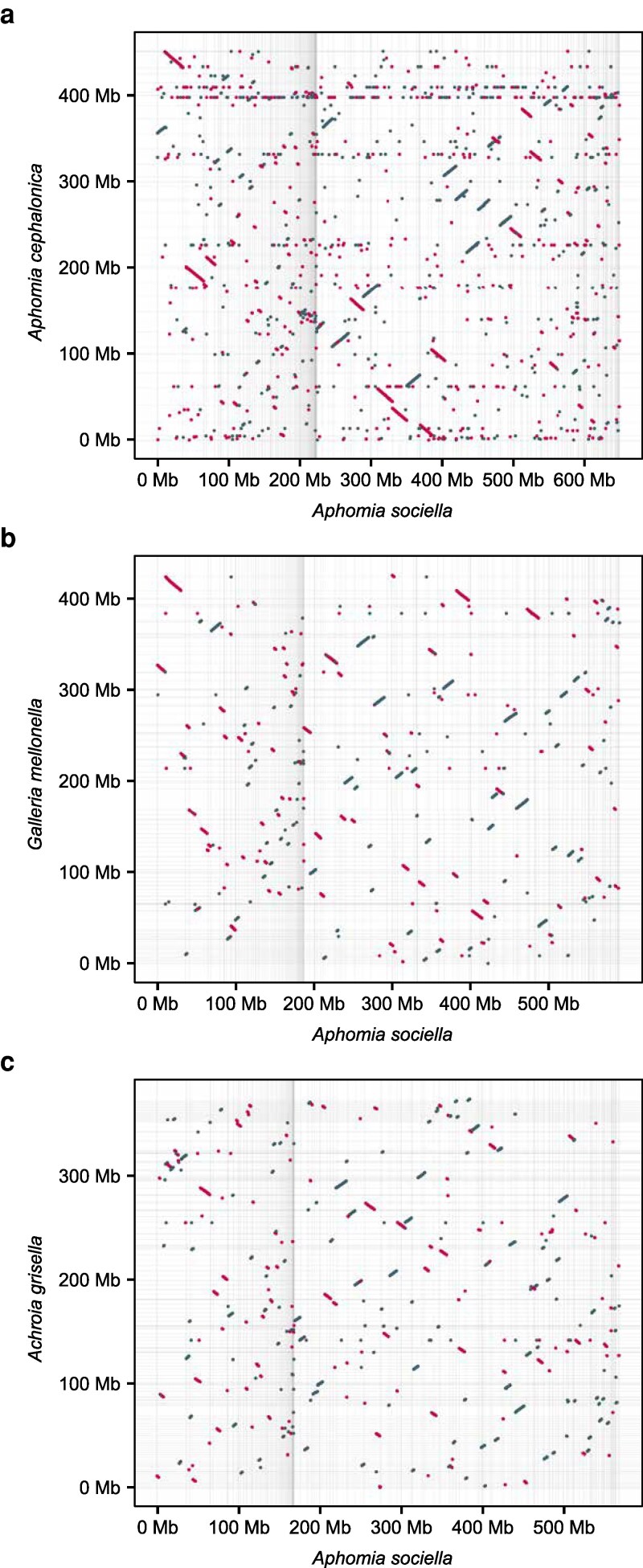
Synteny between the *A. sociella* genome and those of its closest relatives, a) *A. cephalonica* (GCA_040436485.1), b) *G. mellonella* (GCA_027563975.1), and c) *A. grisella* (GCA_030625045.1). Gridlines represent the boundary between contigs in the genome assemblies.

Minimap2 was used to map the whole genome of *A. sociella* to its closest relatives in the *Galleriinae* subfamily. The syntenic blocks are generally less conserved between *A. sociella* and *G. mellonella* compared with *A. cephalonica* (Fig. 3). Nonetheless, multiple contiguous syntenic blocks were identified across the genomes. Notably, the alignment between the genomes of *A. sociella* and *A. cephalonica* revealed longer and more continuous syntenic blocks between the genomes, indicating a higher degree of genome structure conservation.

### Genes and pathways

We next explored the genome to identify genes and pathways that may contribute to metabolic and ecological adaptations of *A. sociella*, with a view toward applied outcomes such as biotechnological exploitation and sustainable pest control strategies.

**Fig. 4.**
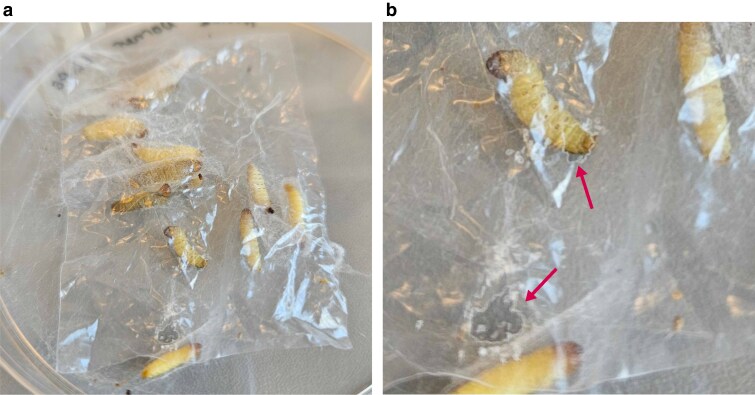
a) *A. sociella* larvae chewing on low-density polyethylene films and b) holes in the polyethylene film made by the larvae.

The larvae of various species within the Lepidoptera order have frequently been observed consuming plastics, such as polyethylene (PE) and polystyrene. Indeed, we confirmed that the larvae of *A. sociella* are also capable of chewing and ingesting PE films (Fig. 4). Such behavior among larvae has attracted considerable scientific interest due to the potential use of these insects in the biodegradation and biotransformation of these recalcitrant synthetic polymers. Although *A. sociella* can penetrate through and consume plastic films, this behavior does not indicate the ability to biodegrade the plastic. Indeed, insects and their larvae eating through plastic film and packaging has long been a problem and has historically been the focus of prevention and control measures (Sreenathan et al. 1960; Cline 1978; Highland 1984; Graham Bowditch 1997).

Notably, in recent years, the larvae of *G. mellonella* have attracted considerable attention due to their ability to ingest PE (Peydaei et al. 2020; Sanluis-Verdes et al. 2022). Two proteins, an insect hexamerin and acidic juvenile-hormone suppressible protein, both of which are found in the *A. sociella* genome (Supplementary Table 3), were proposed to confer *G. mellonella* the ability to oxidize this substrate (Sanluis-Verdes et al. 2022). However, their activity on PE could not be repeated in an independent study (Stepnov et al. 2024), which was in line with the findings of Réjasse et al (2022) who detected no bioassimilation of PE into the tissue of *G. mellonella* larvae when fed ^2^H-labelled PE (Réjasse et al. 2022). A more feasible approach to larval biotransformation of PE could entail a chemical pretreatment step to convert the relatively inert polymer into an oxy-functionalized, low-molecular-weight waxy material that better resembles the natural wax metabolized by these insects. It has indeed been hypothesized that small oxidized aliphatic molecules derived from pretreated plastic can be further metabolized by the insect via the β-oxidation pathway (LeMoine et al. 2020). The genome of *A. sociella* contains genes encoding enzymes involved in the pathway for β-oxidation, including components responsible for the transport of long-chain fatty acids into the cytoplasm and mitochondria (Supplementary Table 3). Multiple copies of cytochrome P450 (CYP) were also identified in the *A. sociella* genome. CYPs are an oxidative enzyme suggested to be implicated in plastic degradation (Son et al. 2024), as they have been linked to the hydroxylation of hydrocarbon components of beeswax (Kong et al. 2019). It is important to note that the β-oxidation pathway and CYP have native functions beyond their potential involvement in plastic degradation, such as oxidizing lipids stored in the fat body of insect larvae (Gao et al. 2023), and oxidation/reduction of organic chemicals (Nauen et al. 2022). It is noteworthy that, for many lepidopteran species including pollinators, CYPs have been shown to play a role in detoxification of secondary metabolites produced by plants and play a role in host adaptation (Heidel-Fischer and Vogel 2015). The exact role of the CYPs identified in the genome of *A. sociella* remains to be elucidated through biochemical characterization on plastic substrates.

Pheromone signaling is an important aspect of the mating behavior of *A. sociella*, and the configuration of many sex pheromones has been characterized for this species (Wallin et al. 2020). The *A. sociella* genome contains genes encoding odorant-binding proteins, chemosensory proteins, odorant receptors, and sensory neuron membrane proteins (Supplementary Table 4), which are most likely involved in the pheromone signaling between the moths (Pregitzer et al. 2014; Jiang et al. 2021). Understanding the underlying mechanisms involved in pheromone synthesis and olfaction can serve as a way to disrupt mating, thus acting as a pest control measure (Kong et al. 2014).

Toll-like receptors (TLRs) are essential components of signaling pathways that regulate both developmental processes and innate immune responses in insects. The *A. sociella* genome encodes multiple TLRs, including proteins predicted to contain both toll/interleukin-1 receptor homology (TIR) domains and leucine-rich repeat (LRR) domains. Insect TLRs are typically characterized by the presence of TIRs and LRRs (Imler and Zheng 2003), and all the 14 putative TLR proteins contain his conserved domain architecture (Supplementary Table 5). Strategies to control moth population, for instance the spongy moth (*Lymantria dispar*), include the use of “bioinsecticides” (Zhang et al. 2019 ), which are designed to disrupt the immune systems of the insect. Therefore, characterizing the presence and structure of TLRs in the bumblebee wax moth is essential for informing the development of effective bioinsecticide-based strategies.

### Conclusion

Here, we present a high-quality contig-level genome assembly of *A. sociella*. A combination of short- and long-read transcriptomic data was utilized to achieve comprehensive and accurate functional gene annotation. This enabled the identification of genes involved in fundamental biological processes as well as those associated with species-specific behaviors, providing valuable insights into the molecular basis of *A. sociella* biology. As expected, phylogenomic analysis placed *A. sociella* in the same clade as *A. cephalonica* and indicated close evolutionary relationships with the other two species in the subfamily *Galleriinae*, namely *A. grisella* and *G. mellonella*. We expect that the availability of an annotated genome for *A. sociella* will facilitate further research into the life cycle and behavior of the moth and can be used in future research on pest control measures. In addition, the genome sequence will facilitate microbiome studies by enabling the effective removal of contaminating host DNA in metagenomic analyses.

Notably, while this work was under revision, a new *A. sociella* genome assembly (GCA_965363045.1) from a single organism collected in the United Kingdom became publicly available. This assembly is at the chromosome level, comprising 305 contigs with a contig N50 of 8.3 Mb and a total genome size of 681.7 Mb. These metrics are comparable to our genome assembly, which consists of 348 contigs with an N50 of 5 Mb and a total genome size of 675 Mb. Importantly, our assembly provides comprehensive annotations, and, together with the newly released genome from a different geographical area, can offer complementary insights to enhance understanding of *A. sociella* genomics and functional biology.

## Supplementary Material

jkaf281_Supplementary_Data

## Data Availability

The Nanopore whole-genome sequencing raw data are available through the Sequence Read Archive (SRA) with the accession number PRJNA1276471. The genome assembly and annotations are available through the NCBI GenBank database with the accession number GCA_052324605.1. Genome annotations can also be found on GitHub at https://github.com/ronjasan/asociella_genome/tree/main/Annotation. The RNA sequencing reads are available through the SRA with the following accession numbers: SAMN49327582 (Illumina short-read RNAseq) and SAMN49327583 (Nanopore long-read RNAseq), both used to annotate the genome assembly. All codes associated with this manuscript are available on GitHub (https://github.com/ronjasan/asociella_genome). Supplemental material available at G3 online.
