# Supplementary material for: Whole-genome assembly and annotation of the bumblebee wax moth, Aphomia sociella
Source: G3 (Bethesda). 2025 Nov 27;16(2):jkaf281. doi: 10.1093/g3journal/jkaf281 (PMC12869067; doi:10.1093/g3journal/jkaf281)
Supplement: jkaf281_Supplementary_Data [file jkaf281_supplementary_data.zip › Supplemental_Material_Legends_G3-2025-406283.docx]

# Supplementary tables

**Table S1:** Statistics for Canu assemblies and the polished genome.

**Table S2:** Available reference genomes for species in the order Lepidoptera used for phylogenomic analysis, together with quality metrics. The species marked in red is from this study.

**Table S3:** Genes hypothesized to be involved in plastic catabolism.

**Table S4:** Pheromone genes.

**Table S5:** Immune genes.
